# Polycrystalline silicon PhC cavities for CMOS on-chip integration

**DOI:** 10.1038/s41598-022-21578-6

**Published:** 2022-10-12

**Authors:** S. Iadanza, G. C. R. Devarapu, A. Blake, P. Acosta Alba, J.-M. Pedini, L. O’Faolain

**Affiliations:** 1 Tyndall National Institute, Lee Maltings, Dyke Parade, Cork, Ireland; 2 Munster Technological University, Rossa Avenue, Bishopstown, Cork, Ireland; 3 Université Grenoble Alpes, CEA, LETI, 38000 Grenoble, France

**Keywords:** Microresonators, Photonic crystals, Silicon photonics, Electronics, photonics and device physics, Electrical and electronic engineering

## Abstract

In this work, we present an on-chip 2D and 3D photonics integration solution compatible with Front End of Line integration (FEOL) using deposited polycrystalline silicon (poly:Si) for optical interconnects applications. Deposited silicon integration on a bulk silicon wafer is here discussed in all its processing steps and configurations. Moreover, results of deposited silicon high-Q Photonic Crystal (PhC) resonators are shown, demonstrating the possibility to employ optical resonators patterned on this material in the next generation of 2D and 3D integrated optical interconnects.

## Introduction

The need for photonics at the CPU level is greatly intensifying in current years. The recent decrease in the size of transistors (down to the 2 nm node equivalent − 333.33 MTr/mm^2^^1,2^ and predicted 1 nm node in 2029^3^), translated into increased transistor densities, has led to a fast growth of the number of floating-point operations (FLOP) that processors can perform, from 1 TFLOP in 2007 to 7.2 TFLOPs in 2015 and to predicted 96.8 TFLOPs in 2022. Moreover, in order to maintain the optimum processor architecture ratio of 1 byte/FLOP, the chip I/O bandwidth needs a continuous scaling over multiple hundreds of Tb/s^1^. However, the chip area is limited to its current size due to manufacture yield and cost, which manifests in a very slow increase in the number of signal pins, greatly limiting chip packaging capabilities. It follows that current bandwidth demands can only be faced by increasing the off-chip clock over 65 GHz by 2029^4^. Concurrently, on-chip heat dissipation limits the maximum chip power consumption to 300 W^2^, with the need to also reduce energy budget for off-chip communication from thousands to tens of fJ/bit. As a consequence of these bandwidth and energy requirements, optical interconnects need to be implemented on the electronics chip in place of existing electrical links. A traditional optics-electronics packaging approach involves the chip-bonding of optical links on the electronics, which is, however, still limited in bandwidth by the pitch of the flip-chip signal I/Os. This approach manifests parasitic electric affecting the performance of both the optical and electronic components, beside imposing a bandwidth density bottleneck. Another promising integration approach consists in the monolithic front-end integration of silicon photonic circuits, which involves the realization of the optical and electronic components in the same SOI layer of crystalline silicon, on a single chip^5^, offering a very compact integration of photonics and electronics, maximizing bandwidth density and lowering parasitic effects. However, beside SOI higher cost compared to bulk Si wafers, this approach severely hinders the performance of electronics as, at telecom wavelengths, low-loss optical confinement in the photonic waveguides requires at least a 1 µm thick buried oxide, while SOI transistors need very thin buried oxide (100 nm or lower) for thermal dissipation and electrostatic effects. Thick buried oxide means that transistor gate lengths must be longer than 100 nm and transistor density decreases^6,7^, considerably limiting processors performance and scalability. Some effort has also been directed towards the front-end integration of waveguides on bulk-Si^8–10^ and thin-SOI substrates^11,12^, but these techniques always comprise the fabrication steps involving the modification of the silicon electronics layer. Another integration approach, referred to as monolithic back-end integration^13^, involves the realization of the photonic components on different plane respect to the electronics layer, offering high bandwidth densities similar to front-end processes, but with added the possibility to keep the optimized fabrication of the transistor layer unchanged, as opposite to front-end integration requirements. The photonic layer typically involves deposited silicon in various phases and forms, as standard crystalline silicon cannot be deposited with standard CMOS technology, but only formed through epitaxial growth^14^ if a crystalline seed is already present, or transferred from a donor c:Si wafer to another target wafer through ion implantation and wafer bonding^15,16^, like in the case of SOI. Deposited silicon materials for photonics can assume the form of silicon nitride (SiN), amorphous silicon (a:Si) and more recently polycrystalline silicon (poly:Si). Despite the optimization of the optical properties of these deposited materials, SiN and a:Si platforms intrinsically exhibit poor electrical properties (low effective carrier mobility) due to their amorphous atomic structure, as opposite to what is typically required for electro-optic modulation, switching, and photodetection. Conversely, deposited poly:Si, still compatible with front-end integration, is characterised by electrical properties similar to monocrystalline silicon, unlocking the possibility to employ this material in fully integrable and very efficient optoelectronic components. Yet, deposited poly:Si normally exhibits high surface roughness and numerous grain boundaries that greatly affects the optical performances of photonic components, mainly due to scattering mechanisms. Moreover, almost all of the reported poly:Si layers required high temperature deposition, annealing and post-treatment (typically T ≥ 900 °C), as shown in^17–22^. This temperature range is not compatible with back-end fabrication processing^23^, leading to electronic doping diffusion, and therefore cannot be used for vertical integration. In this work, we optimise the deposited poly:Si platform through laser annealing and chemical–mechanical planarization processes for the development of high quality PhC resonators to be employed FEOL compatible on-chip integrated optical interconnects (beside remaining fully compatible with BEOL integration^24^). In this work we also create smooth poly:Si on thick SiO_2_ islands nested into a bulk Si wafer. The created poly:Si regions on the wafer are suitable for photonics without compromising the suitability of the rest of wafer for high performance transistors. The enhancement of the light matter-interaction provided by photonic crystals allows high performance photonics to be created that consume only a small fraction of the wafer area.

The manuscript is divided into the following macro-sections:

- The optimisation of the poly:Si deposition, annealing and surface planarization,
- The integration of deposited poly:Si islands on bulk silicon wafers,
- The development of high-Q PhC resonators on poly:Si.

## Fabrication and optimisation of the poly:Si substrates for photonics applications

**Figure 1:**
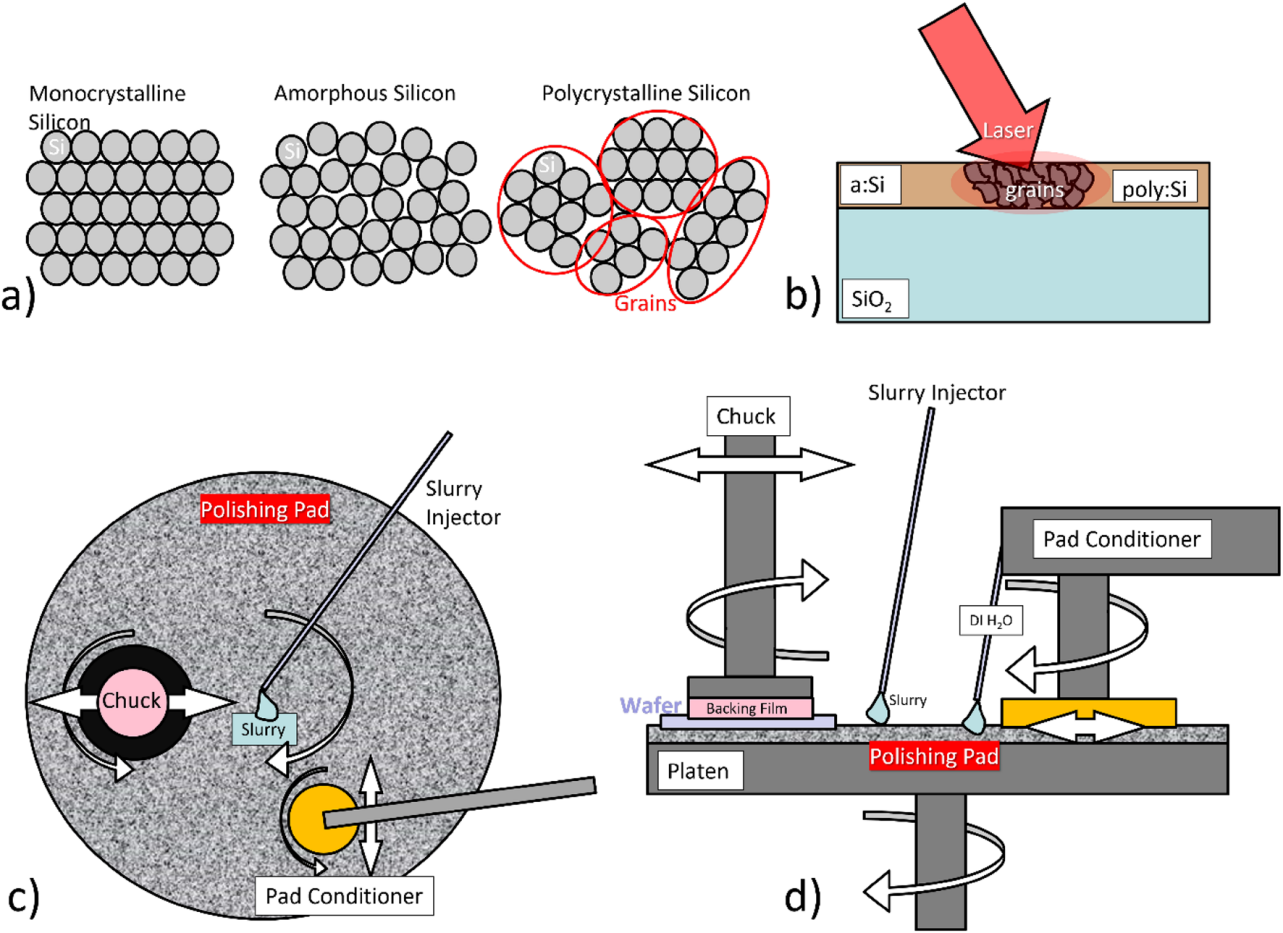
(**a**) Schematics of atomic arrangements in monocrystalline, amorphous and polycrystalline Silicon, (**b**) schematics of the laser annealing process, (**c**) schematics of the CMP process (top view) and (**d**) schematics of the CMP process (side view).

Deposited silicon is one of the most important solutions for 3D integrated Silicon Photonics. However, the material in both the amorphous and polycrystalline form has poor optical qualities due to absorption and roughness scattering mechanisms occurring in the as deposited materials. Their employment in integrated photonics applications requires careful material treatment techniques to lower intrinsic material losses related to linear absorption due to Silicon dangling bonds in the amorphous form and light scattering due to surface and grain boundary roughness in the polycrystalline form. In the case of a:Si, losses can be greatly decreased by means of Hydrogen implantation, quenching absorption at telecom wavelength from silicon dangling bonds by the formation of S–H bonds^25^. Poly:Si, in addition, exhibits poor optical performances mainly related to its microcrystalline nature, for which many different crystalline domains, grains (Fig. 1a), are formed during the crystallisation from the deposited amorphous material. Monocrystalline silicon-like optical properties characterise the inside of these grains, but the different orientation of their crystalline plane from grain-to-grain forms physical rough boundaries between the grains that disrupts the propagation of light via scattering. These grain boundaries are also responsible for the high surface roughness of untreated poly:Si, which dominates light propagation losses on poly:Si platforms^26^. For this reason, dedicated CMOS compatible techniques have been developed to optimise the material roughness and grain size distribution, such Chemical–Mechanical Planarization (CMP) and laser annealing(^27–30^) respectively, which were utilised to prepare the poly:Si substrates for the photonics applications discussed in this work.

**Figure 2:**
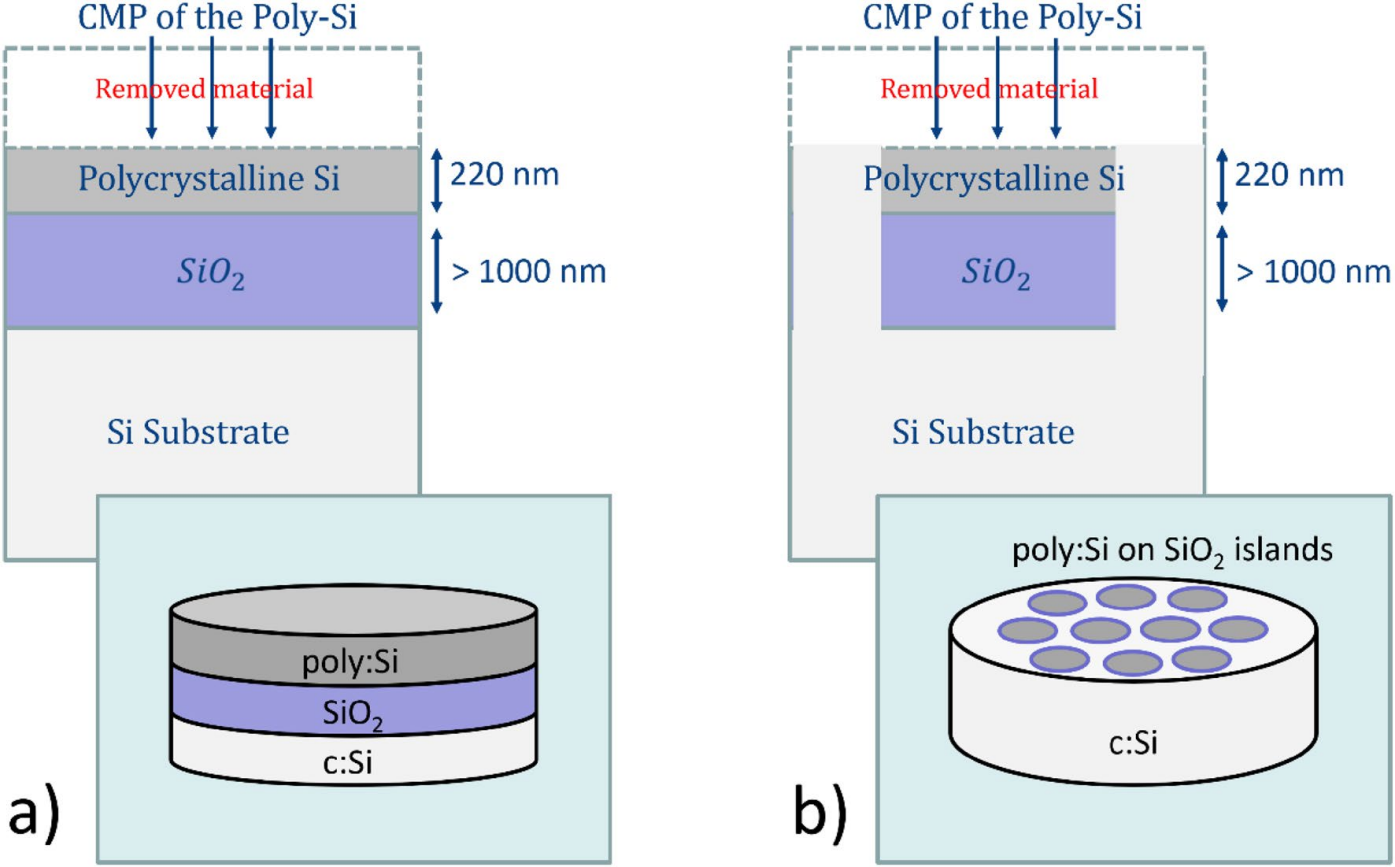
Schematics of the poly:Si substrates in the two configurations: (**a**) thermally annealed poly:Si deposited onto PECVD SiO_2_ for the CMP process optimization and (**b**) poly:Si on SiO_2_ islands nested into a bulk silicon wafer for the fabrication of poly:Si optical resonators.

The schematics of the laser annealing and CMP processes are shown in Fig. 1b,c and d respectively. In this section, the fabrication of the poly:Si substrates for which the PhC cavities have been designed is discussed in detail. The fabrication of the substrates consisted in two different runs carried out at CEA-Leti (Grenoble, France), with the first run focusing on the optimization of the CMP processes on 300 mm wafers (first deposited with a:Si and then annealed into poly:Si—Fig. 2a), and the second focused on the fabrication of the substrates with laser annealed poly:Si on SiO_2_ islands, nested into the bulk silicon (Fig. 2b), on which the actual photonic components are developed. The poly:Si islands were having different sizes ranging from 10 to 1000 µm^2^, therefore able to accommodate one or multiple photonic crystal cavities to be employed as wavelength selective mirrors in hybrid external cavity lasers (HECLs) in vertical coupling configuration^31,32^. As the optical performances of poly:Si strongly depend on surface roughness, which dominates scattering losses, the CMP process had to be optimised to achieve the highest surface smoothness, ideally in the sub-nm range, for the photonic components to be able to have measured Q-factors at least in the 10^3^ range (e.g., minimum values required to get a single-mode PhC-based lasers, as seen in^33^). The substrate fabrication and optimization for optical applications started with the deposition of 2.1 µm of SiO_2_ through means of Plasma Enhanced Physical Vapour Deposition (PECVD) on 300 mm silicon wafers. Subsequently, a layer of amorphous silicon 450 nm thick has been deposited onto the wafers via low temperature ($T=350\,^\circ \mathrm{C}$) PECVD. The thickness of the deposited a:Si layer was higher than the final target of 220 nm as the CMP process removes a good portion of material while polishing the wafer.

**Figure 3:**
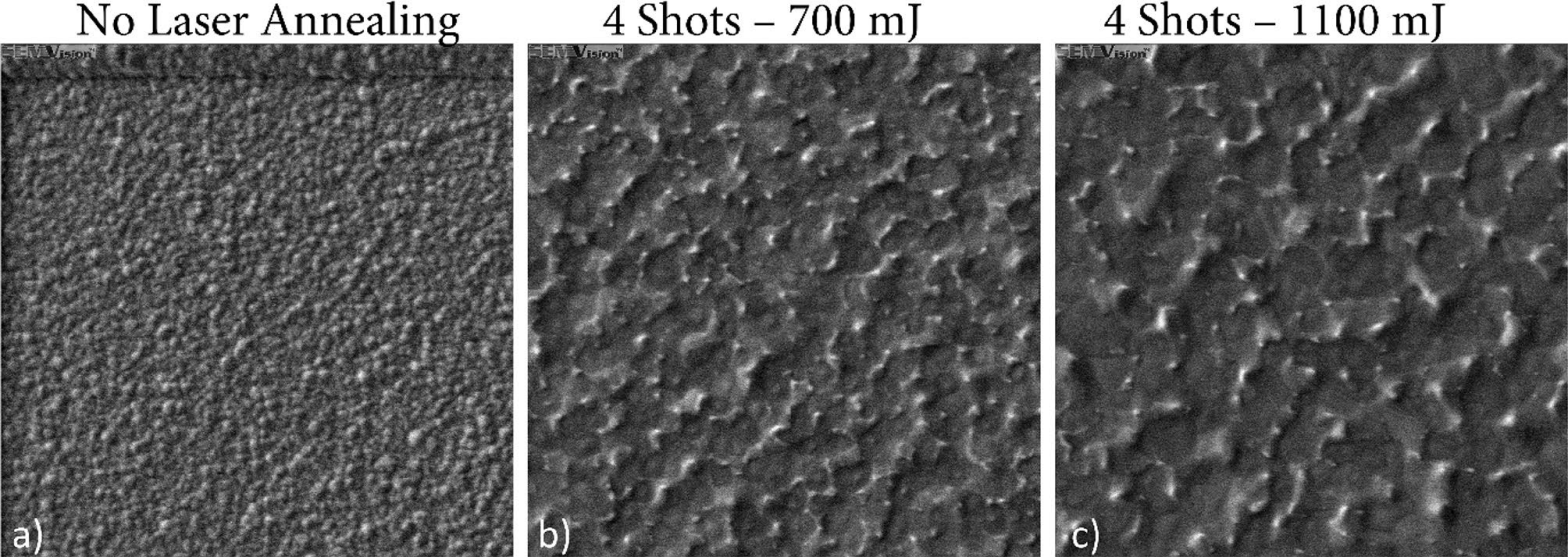
SEM images of the poly:Si substrate: (**a**) before laser annealing (a:Si), (**b**) after 4 laser shots with an energy of 700 mJ and (**c**) after 4 laser shots with an energy of 1100 mJ, highlighting the modification of the poly:Si surface due to the laser-annealing related grain growth.

The a:Si wafers deposited in the first run were thermally annealed to form poly:Si for and were used for the CMP process optimization, while the a:Si on SiO_2_ islands deposited in the second run were treated with pulsed laser annealing while keeping the wafers at a $T=450\,^\circ \mathrm{C}$ to promote poly:Si grain growth at Leti. The poly:Si obtained by these laser annealing steps exhibited grain sizes in the range from tens to hundreds of µm^2^. The growth of the grain sizes of the poly:Si after laser annealing transpires from the SEM images of Fig. 3. This grain size distribution is desirable as entire PhC cavities can be contained in one single grain, therefore avoiding optical interaction with multiple grain boundaries inside the PhC that would increase scattering losses.

The 300 mm wafers polished through CMP in the first run exhibited an average surface roughness of $\{{R}_{a}=0.101 \mathrm{nm}, {R}_{q}=0.049 \mathrm{nm}\}$ with good surface uniformity (centre-edge variation $<\hspace{0.17em}$15 nm), and the final poly:Si layer thickness obtained was in the range of $250\pm 30$ nm.

**Figure 4:**
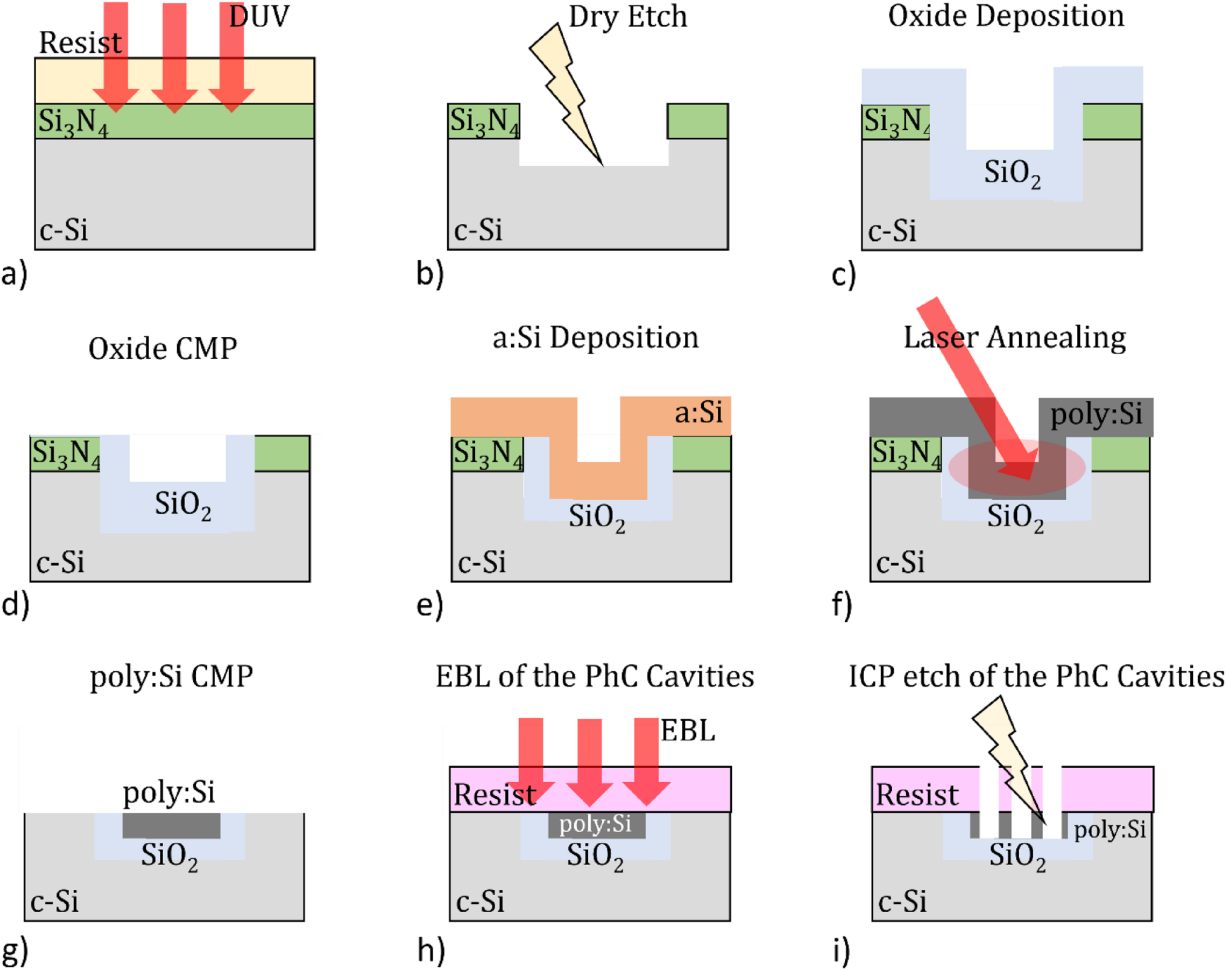
Fabrication workflow of the poly:Si on SiO_2_ islands nested into bulk Si wafers: (**a**) a hard mask of Si_3_N_4_ is deposited and a Deep-UV lithographic step is used to expose rectangles to be used as islands, (**b**) plasma etch of the hard mask and bulk Si, (**c**) PECVD of SiO_2_, (**d**) planarization of the SiO_2_, (**e**) low temperature PECVD of the a:Si, (**f**) laser annealing into poly:Si, (**g**) CMP of the poly:Si, (**h**) Electron-Beam Lithography of the PhC cavities and (**i**) their dry etch.

The fabrication workflow for the poly:Si islands (configuration in Fig. 2b) is more complex than the one for the substrates of Fig. 2a, as area-selective etch and deposition of material is required. The final photonic components (DA and L3 PhC cavities) were then patterned onto the poly:Si islands and measured. The entire fabrication process workflow of the PhC cavities patterned poly:Si on SiO_2_ islands is depicted in the schematics of Fig. 4.

Starting from a 300 mm bulk silicon wafer, a 60 nm thick hard mask of Si_3_N_4_ is deposited through Plasma Enhanced Chemical Vapour Deposition (PECVD) and a 1 µm layer of S1813 photoresist is spin coated on it. A deep-UV lithographic step is performed to expose differently sized rectangular areas that will define the islands (Fig. 4a). A double reactive ion etch (RIE) step first in SF_6_:CHF_3_ chemistry for the Si_3_N_4_ mask and bulk Si to achieve an etch depth greater than 1 µm (Fig. 4b) in which a 1.4 µm thick layer on SiO_2_ is deposited through PECVD (Fig. 4c). Planarization of the SiO_2_ is then followed via standard oxide CMP (Fig. 4d) to achieve a flat oxide filled trenches to be filled with the deposited silicon. A low temperature (T=350 °C) PECVD step is used to deposit 450 nm of a:Si (Fig. 4e), which is then annealed into poly:Si via a pulsed excimer laser source while maintaining the substrate at $T=450\,^\circ \mathrm{C}$ to promote grain growth up to tens of µm^2^ (Fig. 4f). The newly developed poly:Si CMP process is then performed to level and polish the poly:Si islands to the target thickness of $260\pm 40$ nm and sub-nm surface roughness (Fig. 4g). After inspection of the substrates through AFM and SEM to verify surface uniformity and final poly:Si thickness, a 500 nm thick layer of ZEP 520A resist was spin-coated onto the wafer and the specifically designed PhC cavities exposed on it (Fig. 4h) via electron beam lithography (EBL) with a 100 kV system (Elionix ELX100), making sure to align the photonic components to the poly:Si islands. Finally, the patterns were transferred onto the poly:Si via Inductively Coupled Plasma (ICP) etching in N_2_:Cl_2_ chemistry (Fig. 4i). The final poly:Si layer properties and CMP process duration is detailed in Table 1.

**Table 1:** CMP process times and final poly:Si surface properties. S1, S2, S3, S4, S5, S6 and S7 represent the seven different wafers processed with the different CMP regimes.

Wafer	S1	S2	S3	S4	S5	S6	S7
CMP Process P1: VP5000/FSL1531	135 s	130 s	45 s + 90 s	129 s	135 s	135 s	160 s
CMP Process P3: IK2010H/PL6116	–	–	–	60 s	30 s	–	–
Thickness (Max)	273 nm	234 nm	244 nm	237 nm	246 nm	223 nm	221 nm
Thickness (Min)	264 nm	217 nm	228 nm	232 nm	217 nm	200 nm	200 nm
Thickness (Range)	8 nm	17 nm	16 nm	15 nm	20 nm	23 nm	21 nm
Poly:Si Uniformity	35 nm	14 nm	15 nm	14 nm	15 nm	15 nm	15 nm
Roughness (R_a_)	0.185 nm	0.154 nm	0.144 nm	0.132 nm	0.128 nm	0.133 nm	0.131 nm
RMS (R_q_)	0.084 nm	0.071 nm	0.070 nm	0.064 nm	0.063 nm	0.064 nm	0.062 nm

CMP process P1: VP5000/FSL1531 and CMP process P3: IK2010H/PL6116 represent subsequent Chemical–Mechanical Planarization steps with different parameters: wafer chuck pressure, polishing pad speed, conditioning.

**Figure 5:**
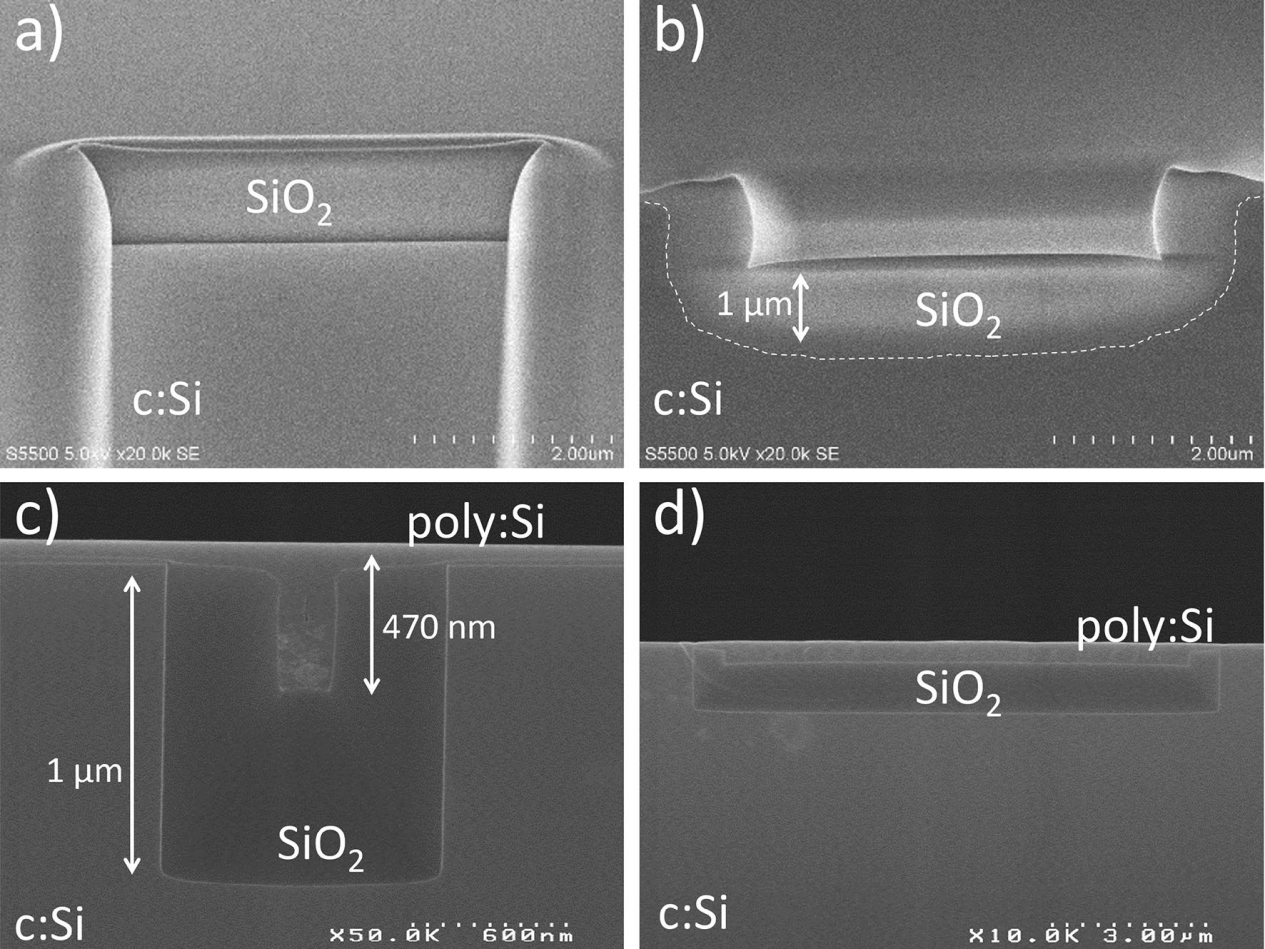
SEM images of: (**a**) deposited SiO2 in the bulk Si trenches (top view), (**b**) deposited SiO_2_ (cross section), (**c**) deposited a:Si (side view) and (**d**) annealed a:Si into poly:Si (cross section).

The deposition of SiO_2_ in the bulk Si trenches and the subsequent deposited and annealed a:Si into poly:Si is shown in the SEM images of Fig. 5.

The top and cross section view of the deposited silica is depicted in Fig. 5a and b respectively, while the deposited a:Si is shown in side-view in Fig. 5c and the annealed a:Si into poly:Si is shown in Fig. 5d, just before the CMP steps. Of great importance is the SiO_2_ thickness being greater than 1 µm, required to well confine the optical modes of the PhC cavities in the poly:Si photonic layer, avoiding evanescent coupling to the bulk Si.

**Figure 6:**
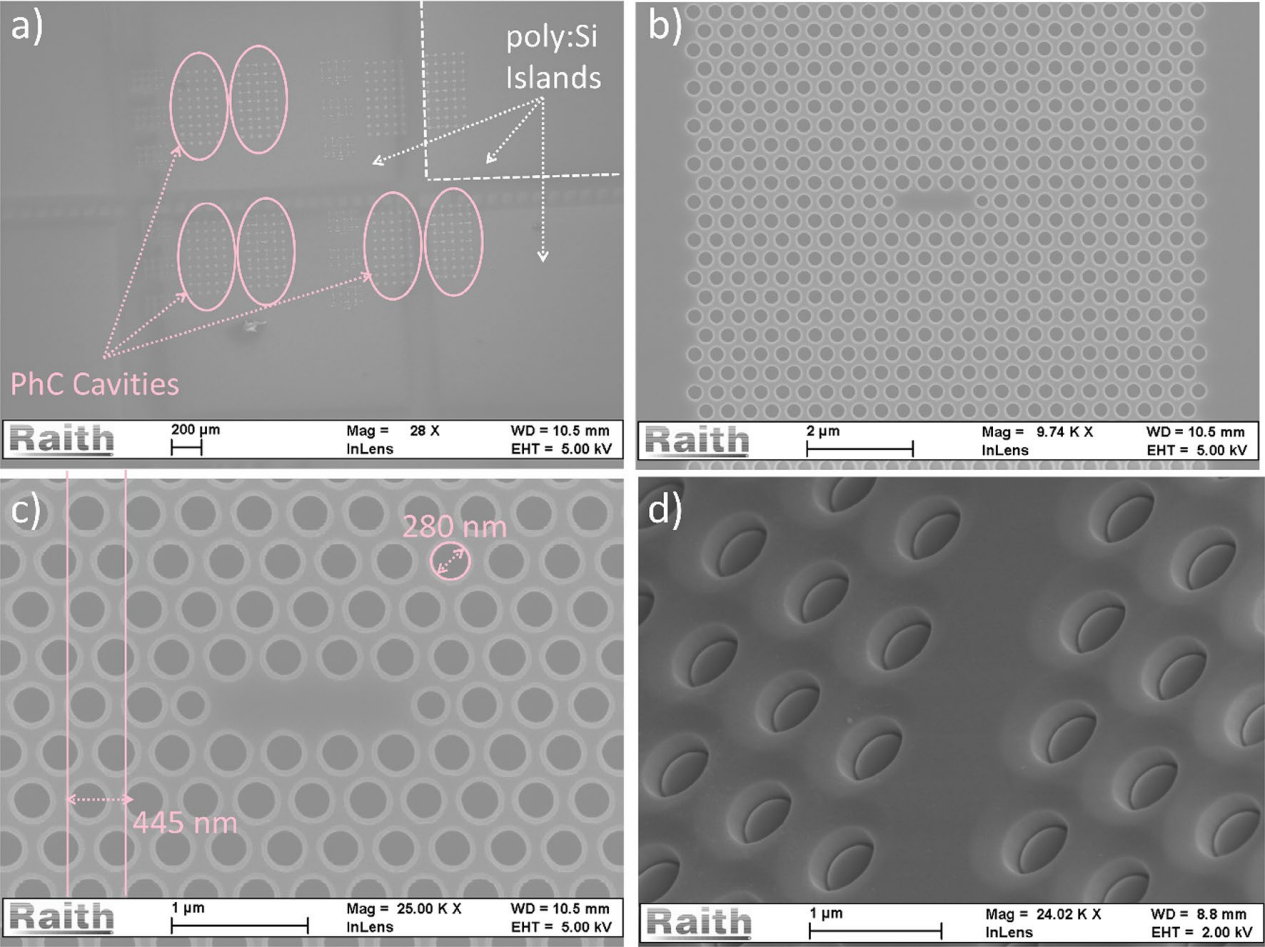
SEM images of the PhC cavities patterned on the deposited poly:Si islands at different magnifications: (**a**) 28x, the PhCs are highlighted by the pink ellipses and dashed arrows and the poly:Si islands are delimited by the white dashed lines, (**b**) Far Field optimized L3 PhC cavity at 9740x, (**c**) same cavity at 25000x, with SEM measurements of hole radius and PhC periodicity and (**d**) PhC cavity imaged at 45°.

The PhC cavities (DA and L3 designs) fabricated onto the polished poly:Si islands are instead shown in the SEM images of Fig. 6. The poly:Si on SiO_2_ islands are visible in Fig. 6a, as shade of a different grey compared to the bulk Si (highlighted with the white dashed lines), while the PhC cavity sets are indicated with the pink dashed arrows. High magnifications of a PhC cavity in one of those sets are shown in Fig. 6b and c, while Fig. 6d shows the microcavity imaged at a $45^\circ$ angle.

The next section will discuss the numerical optimization of the poly:Si cavities and their experimentally measured optical performances.

## Poly:Si photonic crystal cavities numerical optimization

The PhC cavities fabricated on the poly:Si islands have been simulated and optimised for the final poly:Si layer thickness range from 240 to 270 nm (obtained after the CMP processing) via Finite-Difference Time-Domain (FDTD) method with the Ansys–Lumerical software. Hole diameter (d) and lattice period (a) parameters of the 2D photonic crystal cavity design have been optimized for the thickness values of 240, 250, 260, 270 and 280 nm, in order to have designs with high optical performances spanning all the experimental layer thickness range. DA and L3 2D PhC cavity designs have been chosen for the numerical simulations. The central cavity holes shift of the fabricated devices have been optimised to achieve relatively high Q-factor while maintaining a good portion of light coupling in and out of the PhC cavity along its normal direction, as in the far field optimization investigated in^22^. Despite intrinsically lowering their Q-factors, the far field optimisation is required for these cavities in order to measure their response without the need of waveguides connected to them.

**Figure 7:**
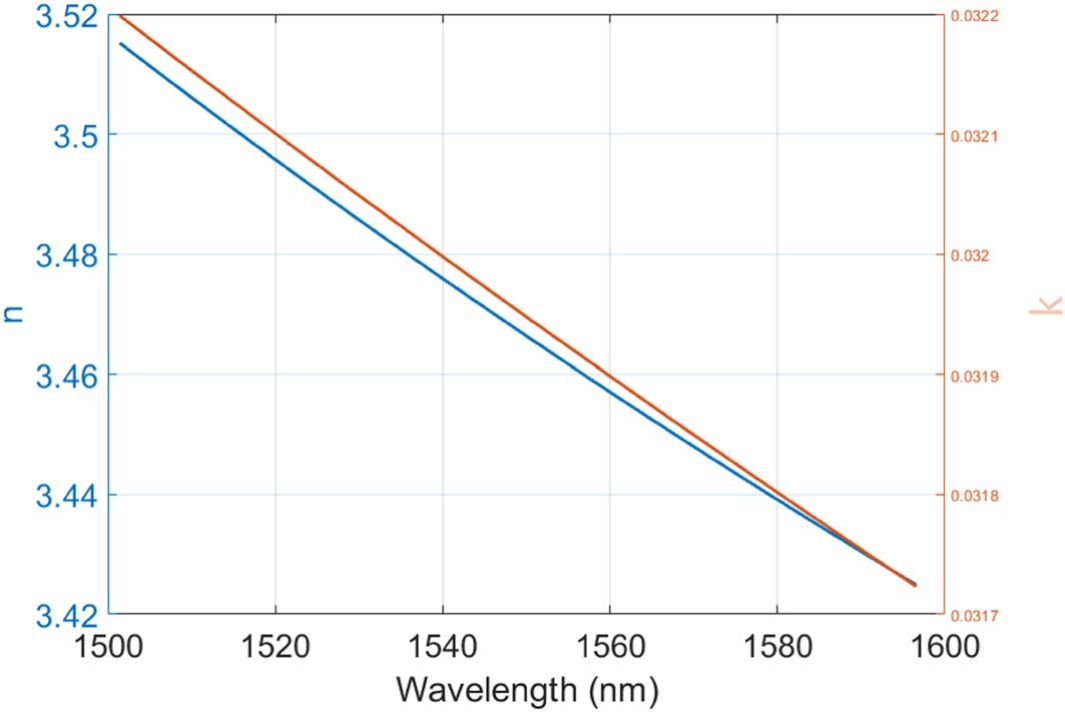
Plot of the real (n) and imaginary (k) refractive index parts of a polished poly:Si wafer against wavelength, measured with the ellipsometer and fitted with a Cauchy model.

In order to accurately simulate the fabricated poly-Si material, the refraction and absorption optical constants n and k (refractive index and extinction coefficient, respectively) of the polished poly:Si wafers have been measured through optical ellipsometry (J.A. Wollam ellipsometer) over a broad range of wavelengths, from 600 to 1600 nm, and the measured values have been imported into the Lumerical material archive, in order to take absorption losses into account for the calculations. The measured ellipsometry data of the poly:Si is shown in Fig. 7, in the range of interest (from 1500 to 1600 nm).

Due to the PhC hexagonal lattice, Lumerical simulations have all been performed with a mesh size of $dx=a/25$ and $dy=a\left(\sqrt{3}/2\right)/25$ and $dz=t/10$, with $t$ representing the poly:Si thickness, in order to have an integer number of cells along each axis.

**Figure 8:**
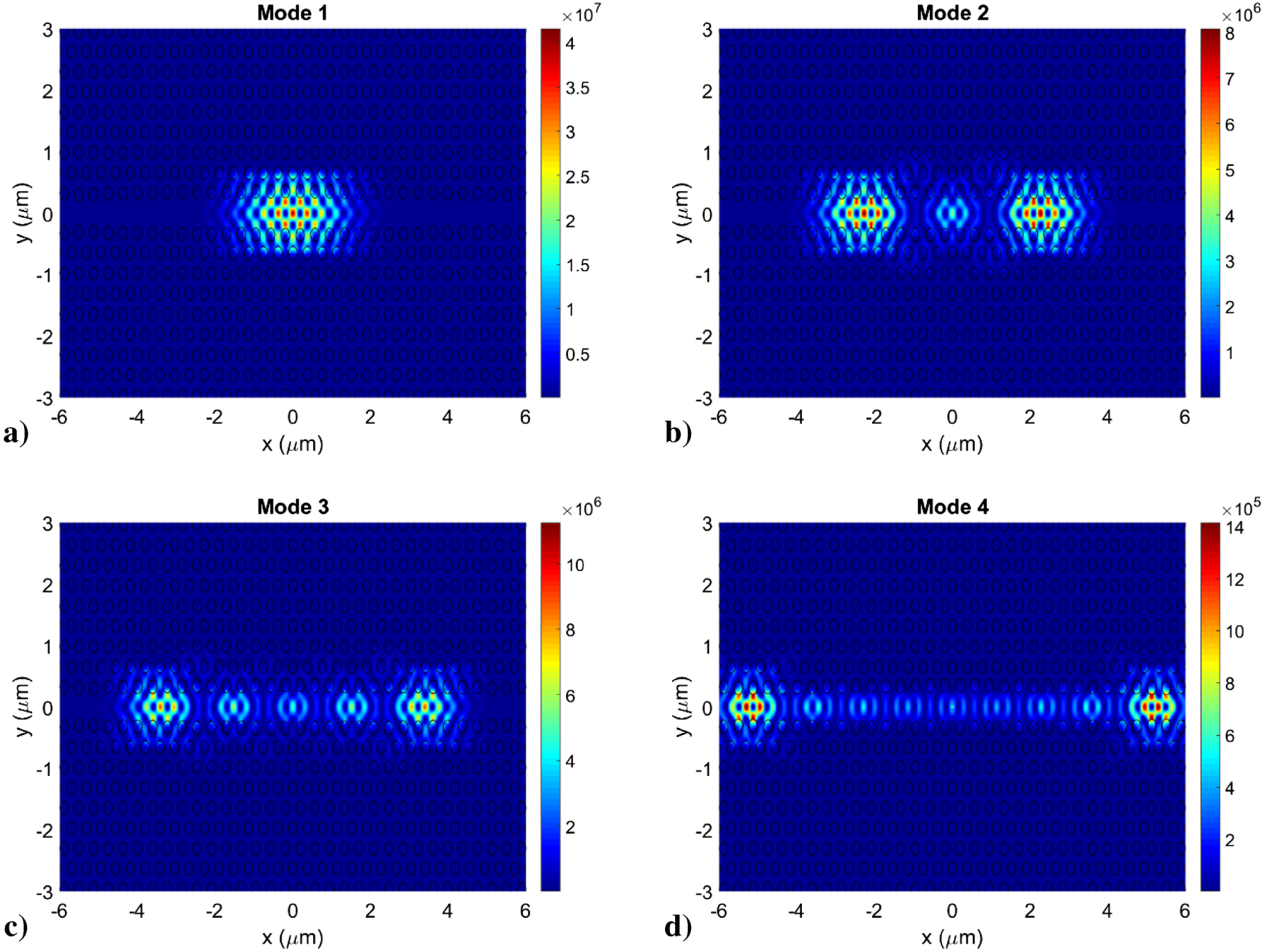
Electric field intensity plot in the xy plane of the first four optical modes confined in a 270 nm thick poly:Si DA PhC cavity. (**a**) Mode 1 (the fundamental mode) and higher order modes (**b**) Mode 2, (**c**) Mode 3 and (**d**) Mode 4.

The calculated optical modes confined in a 270 nm thick poly:Si DA cavity are shown in Fig. 8, in which a colormap of the electric field intensity of the cavity modes is plotted in the xy plane. In the different panels of the figure the fundamental mode (Mode 1) is exhibiting the typical shape with a single intensity peak and no lobe, while the higher order modes (Mode 2, 3 and 4) are showing increasing number of peaks depending on their increasing mode order. Mode 1, 2, 3 and 4 exhibited simulated Q-factors of 5.2·10^6^ at 1578.35 nm, 3.1·10^5^ at 1559.63 nm, 1.7·10^4^ at 1568.90 nm and 6.1·10^3^ at 1530.41 nm.

**Figure 9:**
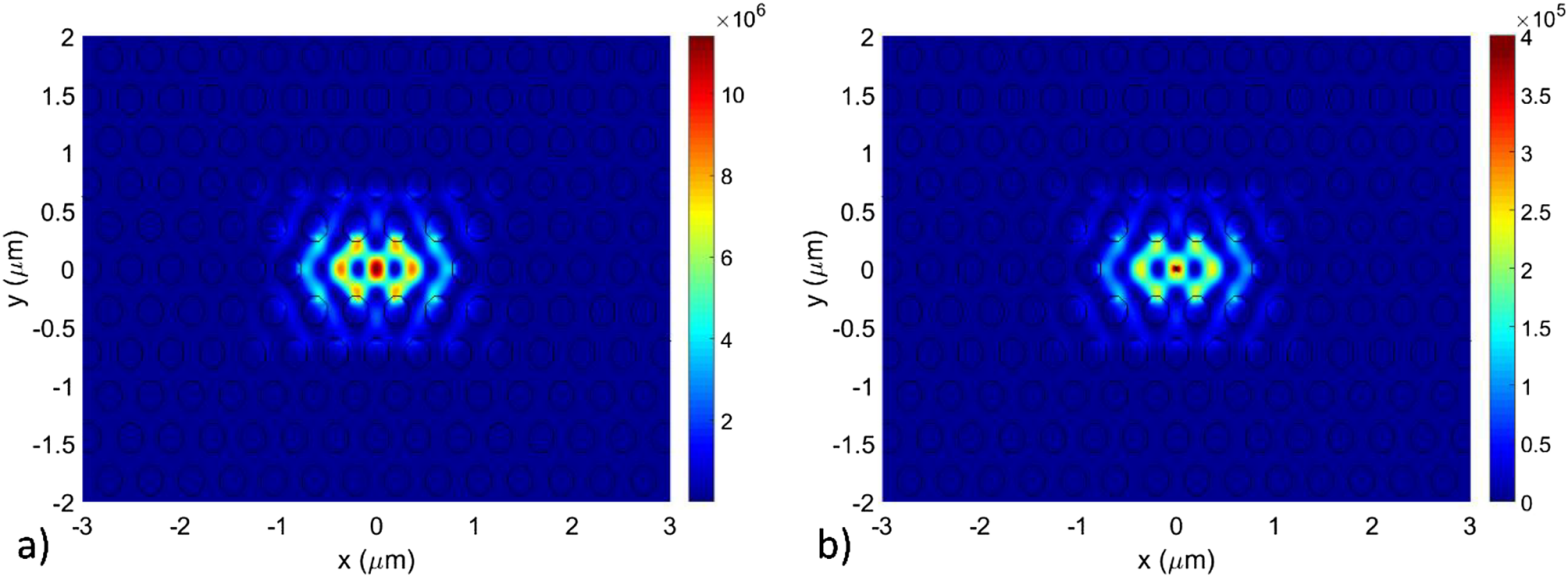
Electric field intensity plot in the xy plane of the first four optical modes confined in a 270 nm thick poly:Si L3 PhC cavity. (**a**) Mode 1 (the fundamental mode) and (**b**) higher order mode (Mode 2).

The simulations were repeated for L3 type PhC cavities for the different deposited Silicon thicknesses and Fig. 9 shows the first confined optical modes in such a cavity with 270 nm thickness. The fundamental mode (Mode 1) has a calculated Q-factor of 5.2·10^5^ at 1538.08 nm while the higher order mode (Mode 2) a calculated Q-Factor of 4.1·10^4^ at 1539.43 nm.

## Measurement of the fabricated PhC cavities on poly:Si

**Figure 10:**
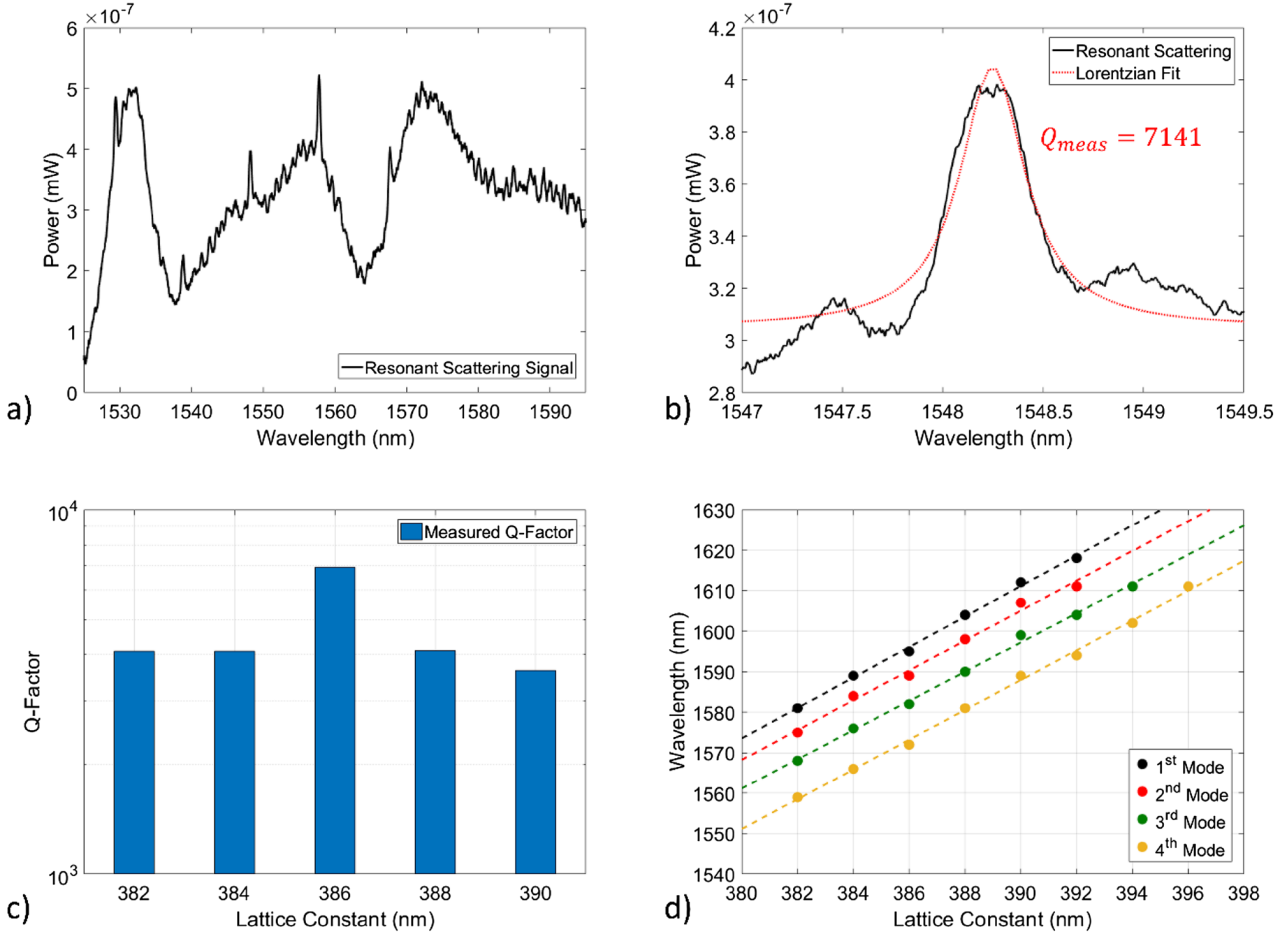
**(a**) Optical spectrum of an L3 PhC measured through resonant scattering technique, (**b**) Spectrum of the measured fundamental mode of (**a**) in which the red curve represents a Lorentzian fit of the resonance, (**c**) Measured Q-factors of L3 cavities with lattice constant increasing with a 2 nm step, (**d**) Resonance wavelength of the optical modes of the L3 PhC cavity versus lattice constant, in which different colours represent different measured resonances.

The far-field optimized dispersion adapted (DA)^34^ and L3 PhC cavities patterned on the polished poly:Si on SiO_2_ islands nested into the bulk Si wafers through mean of EBL and Dry Etching have been optically characterized through Resonant Scattering technique^35^ and the results are summarised in Fig. 10.

Figure 9a depicts the measured optical spectrum of an L3 PhC cavity patterned on a polished 270 nm thick poly:Si island, in which the confined optical modes manifest as intensity peaks spiking out of the source baseline signal as expected with Resonant Scattering technique. A close-up of the measured fundamental mode is shown in Fig. 9b and fitted with a Lorentzian curve with a FWHM of 0.216 nm and peak central wavelength of 1548.31 nm, which leads to a measured Q-factor of 7141. The plot of measured Q-factors of the same L3 PhC cavity design with increasing lattice constant ($a$) is shown in Fig. 9c, in which the lattice constant increases with a step of 2 nm. All the measured Q-factors fall in the range ${10}^{3}-{10}^{4}$ In Fig. 9d, the measured resonance wavelengths of the different cavity modes of the L3 PhC cavity design are plotted against increasing lattice constant (parameter swept with a 2 nm increment in the fabricated devices), showing a very linear behaviour for all the measured optical modes and leading to the possibility of a fairly accurate control of the resonance wavelength through lithographic tuning.

These results demonstrate the possibility to have relatively high-quality optical resonators patterned onto 3D integrated island of poly:Si on SiO_2_ embedded on bulk Si wafers for next generation optical interconnects. The photonic crystal resonator located in the silicon islanded may be connected to other components via a waveguiding layer position vertically above. A variety of components have been demonstrated in vertically coupled configuration such as photodetectors^36^ modulators^37^ and lasers^32^. Such a configuration is ideally suited to this application as the interconnecting waveguides do not reduce the area available for transistors.

The processing steps used in this work are all standard process in CMOS (e.g., shallow trench isolation can provide the SiO_2_ layer). Following the fabrication of polysilicon islands, CMOS processes can be used to fabrication electronic components and the subsequent layers. Our approach is fully CMOS compatible as subsequent CMOS steps will have no effect on the “photonic islands” This approach thus provides frontend integration of electronics and photonics with minimal disruption to the process flow.

## Conclusions

In this work, the development of a fabrication process for the optimization of optical performances of deposited poly:Si is presented through means of Chemical–Mechanical Planarization and Laser Annealing, achieving surface roughness values in the sub-nanometre scale. Fabrication of thin poly:Si on 2 µm thick SiO_2_ islands is achieved on bulk Si wafers, which could operate as photonic layers integrated on the bulk Si electronic layer in photonic-electronic integrated architectures. Moreover, relatively high-Q optical resonators in the form of 2D PhC cavities (DA and L3 designs) have been developed for such poly:Si islands and their optical performances have been measured, with Q-factor values very compatible with the requirements for operation as wavelength selective resonant mirrors in external cavity lasers. This unlocks the possibility to employ deposited poly:Si optical resonators in novel 3D integrated photonic-electronic components for next generation optical interconnects.

## Data Availability

The datasets used and/or analysed during the current study available from the corresponding author on reasonable request. The authors declare no conflict of interest.
